# Outbreak of melioidosis in a highly urbanized area

**DOI:** 10.1080/22221751.2025.2539194

**Published:** 2025-07-24

**Authors:** Kelvin Hei-Yeung Chiu, Xin Li, Shuk-Ching Wong, Jonathan Hon-Kwan Chen, Alan Ka-Lun Tsang, Betsy Wai-Ka Chan, Rosana Wing-Shan Poon, Cyril Chik-Yan Yip, Sally Sau-Man Leung, Simon Yung-Chun So, Tiffany Didik, Michael Yuey-Zhun Ng, Tsz-Yung Hui, Edwin Kwan-Yeung Chiu, Peter Wai-Ching Wong, Hoi-Kei Wong, Sally Cheuk-Ying Wong, David Christopher Lung, Shuk-Kwan Chuang, Albert Ka-Wing Au, Janice Yee-Chi Lo, Kwok-Yung Yuen, Vincent Chi-Chung Cheng

**Affiliations:** a Department of Microbiology, Queen Mary Hospital, Hong Kong Special Administrative Region, People’s Republic of China; b Department of Microbiology, School of Clinical Medicine, Li Ka Shing Faculty of Medicine, The University of Hong Kong, Hong Kong Special Administrative Region, People’s Republic of China; c Department of Health, Centre for Health Protection, Hong Kong Special Administrative Region, People’s Republic of China; d Infection Control Team, Queen Mary Hospital, Hong Kong West Cluster, Hong Kong Special Administrative Region, People’s Republic of China; e School of Nursing, Li Ka Shing Faculty of Medicine, The University of Hong Kong, Hong Kong Special Administrative Region, People’s Republic of China; f Department of Pathology, Queen Elizabeth Hospital, Hong Kong Special Administrative Region, People’s Republic of China; g Department of Pathology, Hong Kong Children's Hospital, Hong Kong Special Administrative Region, People’s Republic of China

**Keywords:** Melioidosis, outbreak, water, *Burkholderia pseudomallei*, water chlorination

## Abstract

In 2022, an outbreak of melioidosis occurred at a highly urbanized district (the SSP district) in Hong Kong. There was a 5-fold increase in cases compared to the average number of cases in the preceding years, with the SSP district accounting for 71.1% of the cases. A total of 568 environmental specimens were collected, including 290 environmental samples and 38 air samples from the freshwater service reservoirs (FWSRs), 16 water samples, and 224 environmental samples from the households of infected cases. *Burkholderia pseudomallei* was cultured from 24 (10.7%) soil samples obtained from the lawn overlying the FWSRs supplying the SSP district or from the surroundings of the air vents used for pressure equalization in the FWSRs. These culture isolates from soil have the same genotype ST-1996 as the patients’ isolates. As *B. pseudomallei* DNA was also detected from the swabs collected from the internal roofs of the FWSRs and inside the water tap at the home of an infected case, the chlorine concentration at the upstream treatment plant outlet was increased from 1 to 1.2 ppm as a precautionary measure. The number and proportion of newly diagnosed melioidosis cases from the SSP district significantly reduced from 32 (71.1%) in 2022 to 6 (35.3%) in 2023 (*p* = 0.018).

## Introduction

Melioidosis is an infectious disease caused by *Burkholderia pseudomallei*. It is highly endemic in Southeast Asia and northern Australia, and likely endemic in many other tropical regions [1]. The disease is primarily acquired through exposure to contaminated soil and water, and can manifest as a wide range of clinical symptoms, ranging from mild localized infections to severe systemic disease [2]. Melioidosis carries a high mortality rate, particularly in individuals with predisposing comorbidities [3].

In endemic regions, melioidosis outbreaks have been frequently associated with heavy rainfall [3]. For example, a study conducted in Singapore examined 550 cases of melioidosis over a 10-year period and found a significant association between incidence and rainfall and humidity levels [4]. Similarly, in Australia, it has been noted that the majority of melioidosis cases (75–85%) occurred during the wet season [5]. Another study conducted in Taiwan suggested that the occurrence of melioidosis outbreaks might be influenced by the combination of a typhoon and unusually heavy rainfall [6]. These findings emphasize the classical association between melioidosis and rainfall, highlighting the importance of climatic factors in the epidemiology.

Previous melioidosis outbreaks have been reported in animals but not in humans in Hong Kong. The earliest recorded outbreak of melioidosis in animals in Hong Kong occurred in an oceanarium, involving dolphins, whales, and seals, in 1975 and 1976 [7]. Here, we report on community case clusters of melioidosis concentrated in a specific geographic area in Hong Kong from August to December 2022, which may not be entirely explained by rainfall variability. We showed a high prevalence of *B. pseudomallei* in soil samples collected from the freshwater service reservoirs (FWSRs) supplying the affected area and demonstrated genetic relatedness between environmental and clinical isolates. Moreover, *B. pseudomallei* DNA was detected in the household environmental sample and from the inside of a water tap from the residence of a patient. The findings of this study contribute to the current understanding of melioidosis epidemiology and stress the importance of proactive surveillance, early detection, and effective control measures to minimize the impact of melioidosis outbreaks.

## Material and methods

### Setting and patient data retrieval

The Centre for Health Protection (CHP) in Hong Kong received reports from the Hospital Authority (HA) of an unusual increase in melioidosis cases since July 2022. Fifteen melioidosis cases, the majority of whom resided in the Sham Shui Po (SSP) district in Hong Kong, were admitted to the hospitals of the same healthcare region from August to October 2022. In view of the significant increase in case numbers compared with the previous years (6–8 from 2017 to 2021), an extensive epidemiological investigation was conducted to identify the potential source of community acquisition of *B. pseudomallei* in defined geographical regions [8]. Patients with any clinical specimen that was culture-positive for *B. pseudomallei* by HA, along with their age, sex, and residential area, were analysed. A new episode of melioidosis is defined as *B. pseudomallei* culture-positive clinical disease occurring ≥ 18 months following initial diagnosis, following the criterion by the Centers for Disease Control and Prevention [9]. The study was approved by the Institutional Review Board of The University of Hong Kong/HA Hong Kong West Cluster (UW 24-198).

### Environmental investigation

In collaboration with the CHP, a retrospective outbreak investigation was conducted to determine the potential source of melioidosis outbreak, which primarily affected the SSP district in Hong Kong. The investigation involved visiting the residential areas of infected patients to assess and collect environmental samples, including water samples and surface swabs. The Water Supplies Department (WSD) facilitated the evaluation of potable water supply from FWSRs to the affected households. During the site visit to the FWSRs, environmental samples, including freshwater and soil samples, were collected.

The sample collection from patients’ homes included swabs of the inside of water taps, drains, washbasins, flushing tanks, and other household water containers, freshwater samples, flushing water samples, surface swabs of the home environment, and sampling of household hygienic products since contamination of disinfectant solutions by members of the *Burkholderia* genus has been well reported [10]. Samples collected at the FWSRs included freshwater samples from the service reservoirs, soil samples from different depths of the overlying lawn and corners of the air vents, and swabs taken from air vents and internal roofs of the FWSRs.

### Environmental sampling

Flexible premoistened sterile polywipe sponge swabs measuring 5 × 10 cm (Medical Wire & Equipment, Corsham, UK) were used for environmental sampling as previously described [11–13]. An air sampler, SAS Super ISO 180 model 86834 (VWR International PBI S.r.l., Milan, Italy), was used to collect 1000 L of air at a rate of 180 L/min for each air sample [14]. The air collected was directly passed onto modified Ashdown's agar (30 g/L trypticase soy broth, 40 mL/L glycerol, 5 mg/L crystal violet, 4 mg/L gentamicin, and 0.05 g/L neutral red) plates during a 6-min process. Air sampling was also performed using modified Ashdown's agar as settle plates with 25-min exposure.

### Microbiological investigation

Environmental samples were processed on the same day of the on-site investigation. The sample processing procedures were all done in a Biosafety Class 2 cabinet to prevent contamination and they were all handling with aseptic techniques. For freshwater samples, 25 mL of each water sample was centrifuged at 2000×g for 10 min and then concentrated to 1 mL. The concentrated sample was used for nucleic acid extraction and microbiological culture. For swab samples, they were each suspended in 3 mL of sterilized phosphate buffer saline (PBS) and vortexed for 5 min. One millilitre of PBS was used for nucleic acid extraction and microbiological culture. For soil samples, about 10 g of soil was transferred into 3 mL of sterilized PBS and was vortexed for 5 min. One millilitre of PBS was used for nucleic acid extraction and microbiological culture. All samples with *B. pseudomallei* detected by PCR and selected samples with negative PCR reactions were processed for bacterial culture. All air samples were processed for bacterial culture only, PCR was not performed.

For bacterial culture of *B. pseudomallei*, 100 μL of each PBS concentrate or swab suspension was inoculated into a 5 mL selective broth (37 g/L brain heart infusion broth, 20 g/L gentamicin, 15 g/L vancomycin, and 1 g/L amphotericin B) and incubated for 48 h in a 35℃ humid incubator. The enriched broth was sub-cultured onto modified Ashdown's agar plates and incubated for 48 h at 35℃. Cultures were examined for growth of *B. pseudomallei* daily and negative plates were re-incubated until day 7 after inoculation. Suspected *B. pseudomallei* colonies were selected for matrix-assisted laser desorption/ionization time-of-flight mass spectrometry (MALDI-TOF MS) (Bruker Daltonics, Bremen, Germany) with the Bruker Security-Relevant (SR) library and we further confirmed the identity with a curated in-house *B. pseudomallei* spectra database [15,16].

For molecular detection of *B. pseudomallei*, nucleic acid extraction was performed using the eMag automated extraction system (bioMérieux, Marcy-l'Étoile, France) with an elution volume of 25 μL. For the removal of PCR inhibitors in the soil DNA extracts, an extra column-based purification step was performed using the QIAquick PCR Purification Kit (Qiagen, Hilden, Germany). Real-time PCR targeting the *ISBp1* of *B. pseudomallei* was modified from the protocol previously described [17]. In brief, single tube nested PCR amplification using the QuantiNova Probe PCR Kit with outer forward primer (5′-AGAGCTTCAACGGCAAGTTCCGAGATGAATGCTT-3′), outer reverse primer (5′-AACTCGTCGGGCGTCAGATAGGCCAGACTCGAAT-3′), inner forward primer (5’-GAGCCTTGAATGGTTTCGAA-3’), and inner reverse primer (5’-CGGTCGGATGGCATTGTA-3) and *ISBp1* specific hydrolysis probe (FAM- ACGGAAGCGAAGGTTGTGATCGAG-3IABkFQ) was performed with the following parameters: an activation and denaturation step at 95℃ for 2 min, first round amplification by 20 cycles of 5 s at 95℃ and 30 s at 72℃, then followed by 40 cycles of 5 s at 95℃ and 30 s at 55℃. The PCR amplification was performed on the LightCycler96 platform (Roche Diagnostics, Mannheim, Germany). A clinical *B. pseudomallei* strain DNA extract and nuclease free water were used as the positive and negative PCR control for each batch of run.

For whole-genome sequencing of bacterial isolates, DNA libraries were prepared using Illumina Nextera XT / DNA Prep library preparation kits (Illumina Inc, CA, USA) according to the manufacturer’s instructions. The libraries were then sequenced on the Illumina MiSeq platform. The sequence assembly was performed by Unicycler v0.5.1 and the core single nucleotide polymorphism (SNP) genome analysis was conducted using Snippy v4.6.0. Phylogenetic tree was constructed using IQ Tree v2.3.6 with TVM + F + ASC and K3P + ASC substitution models. The sequence data presented in the study are deposited in the NCBI database under BioProject PRJNA1271730.

### Weather conditions in Hong Kong

The weather conditions in Hong Kong from 2014 to 2023 were retrieved from the open dataset of the Government of Hong Kong Special Administrative Region (https://data.gov.hk/en-datasets/category/climate-and-weather) and the Hong Kong Observatory [18]. The daily mean temperature, daily mean relative humidity, and daily total rainfall recorded at the Hong Kong Observatory were retrieved and analysed. The monthly meteorological norms for Hong Kong (1991–2020) were also retrieved [19]. A weighted tropical cyclone score modified from the methods described by Wu et al. [20] and Su et al. [21] was calculated by multiplying the duration of signals hoisted (hours) and intensity (tropical cyclone warning signal 1–3: 1 point; signal 8: 3 points; signal 9–10: 5 points).

### Statistical analysis

Statistical analyses were performed using PRISM version 10.2.2 (GraphPad Software, San Diego, CA). The geographical distribution of cases was compared using Fisher’s exact test. The daily mean temperature, relative humidity, and daily total rainfall across the years were compared using Friedman test and the cyclone warning signals were compared using 2-way ANOVA. The daily mean temperature, relatively humidity, and rainfall by month between years 2022 and 2023 were compared using Wilcoxon matched pairs signed rank test. A *p* value of < 0.05 was considered significant. Graphs were created using PRISM, Datawrapper (https://www.datawrapper.de/), and ArcGIS Online (Esri).

## Results

### Epidemiology of melioidosis in Hong Kong

**Figure 1.**
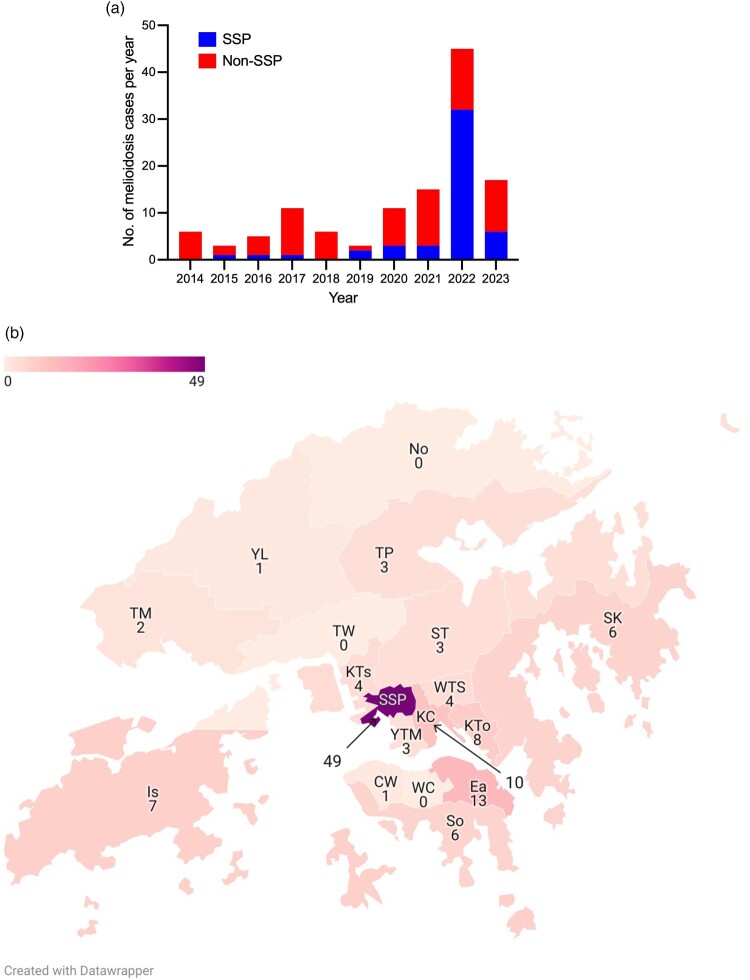
Summary of melioidosis cases in Hong Kong, 2014–2023. (A) Yearly number of melioidosis cases. A new episode of melioidosis is defined as *Burkholderia pseudomallei* culture-positive clinical disease occurring ≥ 18 months following initial diagnosis. (B) Geographical distribution and aggregated number of melioidosis cases in Hong Kong. CW – Central & Western; Ea – Eastern; Is – Islands; KC – Kowloon City; KTs – Kwai Tsing; KTo – Kwun Tong; No – North; SK – Sai Kung; ST – Sha Tin; SSP – Sham Shui Po; So – Southern; TP – Tai Po; TM – Tuen Mun; TW – Tsuen Wan; WC – Wan Chai; WTS – Wong Tai Sin; YTM – Yau Tsim Mong; YL – Yuen Long.

From 1 January 2014 to 31 December 2023, a total of 120 patients had 122 episodes of culture-confirmed melioidosis. Two patients each had two separate episodes of melioidosis as defined above. All patients had at least one clinical specimen that was culture positive for *B. pseudomallei*, with 78 (65.0%) having positive blood cultures and 10 (8.3%) having positive cultures from other normally sterile sites without bacteraemia, including pleural fluid (4), peritoneal fluid (3), pericardial fluid (1), joint fluid (1), and liver abscess drainage specimen (1). The remaining 32 patients had *B. pseudomallei* isolated from non-sterile sites only, including lower respiratory tract (14), wound swab or pus aspirate (10), urine (7), and one with missing site information. Thirty-five (29.2%) were female, and the median age at first isolation of *B. pseudomallei* from any clinical specimens was 69 years (range, 8–95 years). Notably, the number of patients diagnosed with melioidosis in 2022 increased by 5-fold compared with the average number of the preceding years (Figure 1A, Supplementary Table 1). The incidence of melioidosis was also the highest in 2022 (0.60 cases per 100,000 population), taking into account the difference in population size between 2014 and 2023 (Supplementary Table 2).

Between 2014 and 2023, 49 (40.8%) cases of melioidosis resided in the SSP district, followed by the Hong Kong Eastern district (13 cases, 10.8%) and Kowloon City district (10 cases, 8.3%) (Figure 1B). Other districts contributed to fewer than 10 cases each across the years. Calculated using the population profile in 2023 [22], the cumulative incidence was highest in the SSP district (11.3 cases per 100,000 population), followed by the Islands (3.6 cases per 100,000) and Eastern district (2.5 cases per 100,000) (Supplementary Table 3). In 2022 alone, 32 (71.1%) newly diagnosed melioidosis cases were geographically localized to the SSP district, which represented a significant increase compared to the yearly average of 2014–2021 (*p* < 0.0001), as reported previously [20].

### On-site outbreak investigation

In view of the melioidosis outbreak in July–October 2022 with clustering at the SSP district, an outbreak investigation was conducted to look for the potential sources of community exposure. In this investigation, a total of 568 environmental samples were collected. Initial epidemiological investigations by the CHP did not reveal any common places visited by the affected patients [8], and most cases were clustered in part of the SSP district within a radius of about 1 km and lived in different residential buildings. There was no family clustering. Visits were made to the residential addresses of 26 cases residing in the SSP district in October–November 2022. A total of 224 environmental samples were collected from the home environment, including 86 swabs from the inside of water taps, shower heads, washbasins, water drains, flushing tanks, toilet bowls, water filters, and the inner surface of water pipes right before entry to the water metre, 27 freshwater samples from taps and water tanks, 8 flushing water samples, and 12 samples from other household water containers including vase, fish tank, charcoal filters, and misting devices for culture and PCR for *B. pseudomallei* (Table 1). The rest of the samples consisted of 25 surface swabs from home environment, 6 soil samples from in-door plants, and 60 samples of household hygienic products, face masks, and skin care products, following report of melioidosis outbreak associated with aromatherapy spray [23]. *B. pseudomallei* was detected by PCR in the water from a vase and in the swab taken from the inside of a bathroom washbasin tap from the home of one patient, with Ct values of 15.11 and 16.27, respectively, by nested PCR. The estimated bacterial loads were 44.25 and 21.88 copies/mL, respectively. Neither specimen yielded positive culture for *B. pseudomallei*.

**Table 1.** Household samples collected during on-site investigation to the residential addresses of 26 cases residing in the Sham Shui Po district in October–November 2022.

Specimen type	Total number collected	No. (%) positive for *Burkholderia pseudomallei* by PCR
Swabs from the inside of water taps, shower heads, wash basins, water drains, water pipes, flushing tanks, toilet bowls, and water filters	86	1 (1.2%)^a^
Freshwater	27	0
Flushing water	8	0
Other household samples^b^	12	1 (8.3%)^c^
Environmental swab	25	0
In-door soil	6	0
Miscellaneous (household hygienic products, face masks, and skin care products)	60	0
Total	224	2 (0.9%)

^a^ The positive sample was taken from the inside of a bathroom washbasin tap.

^b^ Include water in vase, fish tank, charcoal filters, and misting devices.

^c^ The positive sample was collected from a vase.

In view of the positive PCR result from household environmental samples, the FWSRs supplying potable water to residential buildings of the cases in the SSP district (FWSRs A-E) were inspected (Supplementary Figure 1). The FWSRs are constructed at elevated locations to take advantage of gravity flow. They adopted enclosed design and were constructed with reinforced concrete. The rooftops of the FWSRs were covered with soil and lawns [24]. Air vents with wire gauze mesh filters were installed at the top of the reservoirs and elevated above the ground and functioned to equalize the pressure difference between the outside and the inside of the enclosed concrete reservoir, where negative pressure was generated by water drainage (Supplementary Figure 2). The wire gauze mesh filters were designed to prevent the ingress of large-particle foreign substances.

A total of 344 environmental samples were collected at the FWSRs, including 104 soil samples taken at different depths of the lawn or from the ground (including 2 slurry samples taken directly from the lawn overlying the FWSR), 120 soil samples taken from the 4 corners of 30 air vents, 38 air samples taken by air sampler or settle plate method, 45 swabs taken from air vents, 21 swabs taken from the roof of water storage compartments, 10 freshwater samples, and 6 swabs taken from water taps or sink drains (Table 2). *B. pseudomallei* was detected by PCR in a total of 125 samples, including 119 out of 224 (53.1%) soil samples from the lawn/ground or surroundings of air vents, among which 24 (20.2%) were culture positive for *B. pseudomallei*. The highest detection rates were seen in FWSRs B (63.6% PCR positive from the lawn/ground and 100% PCR positive from the corners of air vents) and C (76.7% PCR positive from the lawn/ground and 85.0% PCR positive from the corners of air vents), the two FWSRs located in the vicinity of the affected area in SSP district (Supplementary Figure 1). The mean Ct value of all nested-PCR-positive soil samples was 12.96 (range, 4.75–20.14). Five surface swabs of air vents at FWSRs B and C, and one swab from the internal roof of FWSR B next to the entry of an air vent were also positive for *B. pseudomallei* by PCR but negative by culture. *B. pseudomallei* was detected by PCR in 2 slurry samples taken from the lawn of FWSR C (classified as soil samples), but freshwater samples taken from the storage compartments all yielded negative results. Air samples collected from the FWSRs were all negative for Burkholderia pseudomallei by culture. The Ct values of PCR-positive environmental samples from FWSRs are summarized in Supplementary Table 4.

**Table 2.** Environmental samples collected during on-site investigation to 6 freshwater service reservoirs within and near the SSP district in October–November 2022.

FWSR	Soil samples from the lawn or ground^a^	Soil collected at corners of air vents	Air samples	Air vent swabs	Roof of FWSR	Fresh water samples	Water tap or sink drain swabs
A	21	40	0	10	0	0	0
PCR +	4 (19.0%)	23 (57.5%)	–	0	–	–	–
Culture +	3 (14.3%)	2 (5.0%)	–	0	–	–	–
B	11	20	12	9	21	5	0
PCR +	7 (63.6%)	20 (100%)	N/A	3 (33.3%)	1 (4.8%)	0	–
Culture +	0	0	0	0	0	0	–
C	30	20	26	5	0	1	0
PCR +	23 (76.7%)	17 (85.0%)	N/A	2 (40.0%)	–	0	–
Culture +	13 (43.3%)	6 (30.0%)	0	0	–	0	–
D	22	40	0	10	0	3	6
PCR +	2 (9.1%)	14 (35.0%)	–	0	–	0	0
Culture +	0	0	–	0	–	0	0
E	20	0	0	11	0	1	0
PCR +	9 (45.0%)	–	–	0	–	0	–
Culture +	0	–	–	0	–	0	–
Total	104	120	38	45	21	10	6
PCR +	45 (43.3%)	74 (61.7%)	N/A	5 (11.1%)	1 (4.8%)	0	0
Culture +	16 (15.4%)	8 (6.7%)	0	0	0	0	0

FWSR: freshwater service reservoir.

^a^ Include 2 slurry samples taken from the lawn of FWSR C.

*B. pseudomallei* were isolated in culture from 24 (10.7%) soil samples, all originating from FWSRs A and C, including one slurry sample taken from the lawn of FWSR C. Phylogenetic analysis using core SNP analysis showed that the environmental strains were closely related to the patients’ isolates from the SSP district, all belonging to the ST-1996 lineage (Figure 2A). No previous reports of an outbreak involving the *B. pseudomallei* ST-1996 lineage were found in other countries prior to the SSP outbreak. Among the ST-1996 cluster, *B. pseudomallei* sequences from 26 out of 33 patient samples (78.8%) in the 2022 SSP outbreak were phylogenetically linked to the *B. pseudomallei* sequences found in environmental samples from FWSR C (Figure 2B). Patient samples from other districts in 2022 were genetically distant from the ST-1996 lineage.

**← Figure 2.**
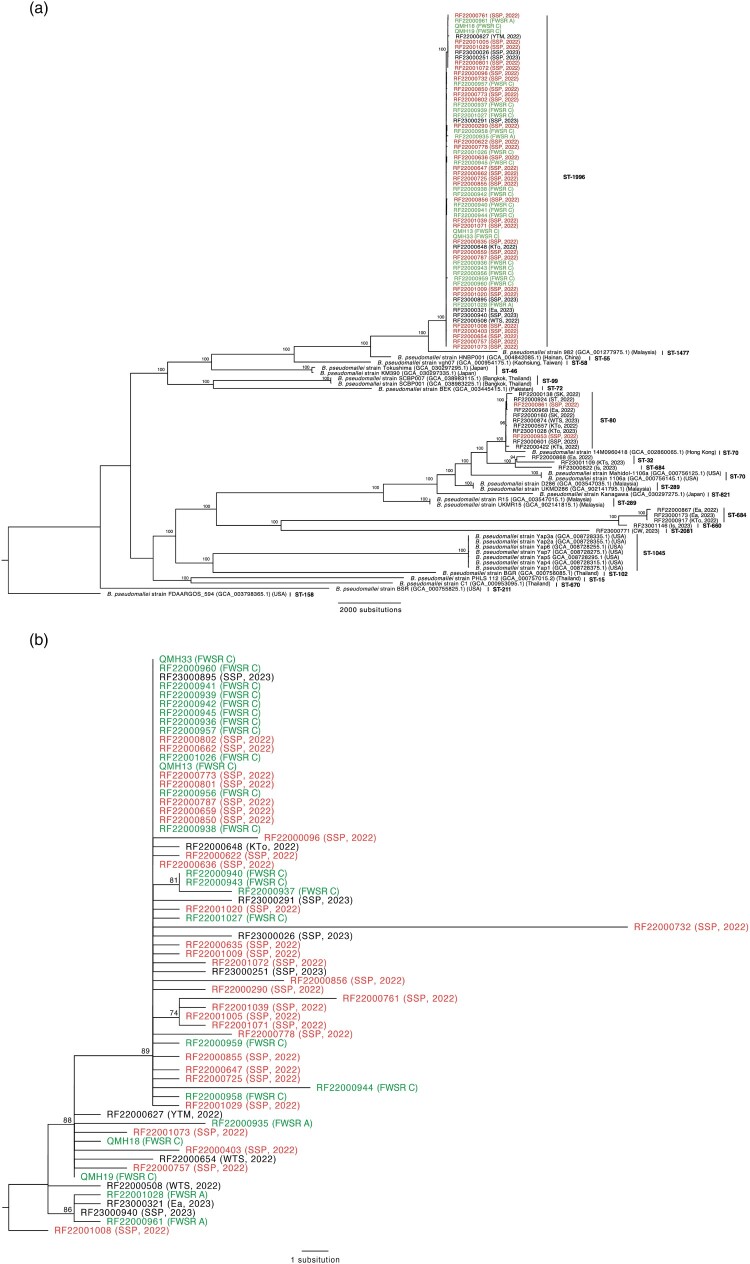
Phylogenetic analysis of patient and environmental isolates. (A) Single nucleotide polymorphism (SNP) phylogenetic analysis of clinical and environmental *B. pseudomallei* isolates in Hong Kong between 2022 and 2023. The *B. pseudomallei* FDAARGOS_594 strain (GCA_003798365.1) (MLST type 158) was used as the outgroup of the alignment. (B) Core SNP genome phylogenetic analysis of the ST-1996 major outbreak cluster. The RF22001008 sequence was used as the reference for the alignment. The colours of the labels indicate the source of sampling: red labels represent clinical strains isolated from patients residing in SSP district in 2022, green labels represent environmental samples collected in SSP district in 2022, and black labels represent either clinical strains isolated from patients residing outside SSP district, samples collected later in 2023, or reference sequences from the NCBI GenBank. Epidemiological information of the isolates, including the location of sampling (CW – Central & Western district, FWSR A – Fresh Water Service Reservoir A in SSP district, FWSR C – Fresh Water Service Reservoir C in SSP district, Ea – Eastern, Is – Islands district, KTo – Kwun Tong district, KTs – Kwai Tsing district, SK – Sai Kung district, ST – Sha Tin district, SSP – Sham Shui Po district, WTS – Wong Tai Sin district, YTM – Yau Tsim Mong district), and sampling year, was mentioned in brackets.

### Recommendation for outbreak control and outcome measurement

**Figure 3.**
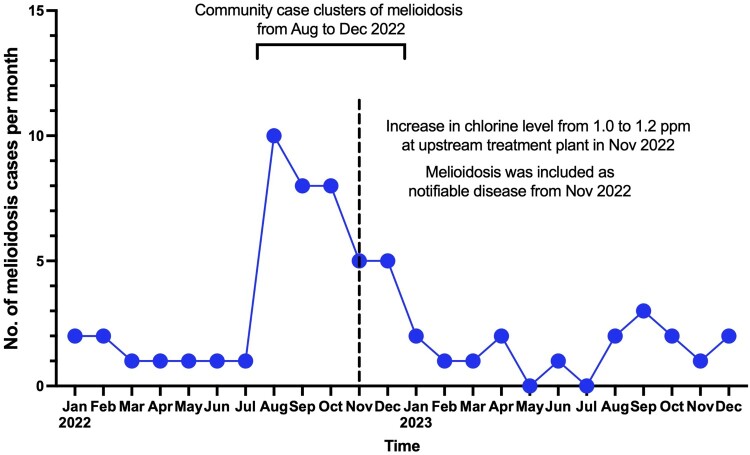
Monthly number of melioidosis cases and public health measures in Hong Kong, 2022–2023.

In response to the finding of positive environmental samples from the FWSRs, the residual chlorine level at the upstream treatment plant outlet was increased to 1.2 ppm in November 2022 as a precautionary measure. Real-time residual chlorine monitoring systems were installed at outlets of FWSRs to ensure that the desired level of chlorination is maintained. High-efficiency particulate air (HEPA) filters were also installed over the air vents to prevent soil particles from entering the FWSRs. Furthermore, melioidosis was included as one of the statutorily notifiable diseases to enhance surveillance starting from 11 November 2022 [25]. With the measures in place, the number of melioidosis cases significantly decreased in 2023 (Figure 1A and Figure 3). Specifically, the number of newly diagnosed melioidosis cases from the SSP district significantly reduced from 32 (71.1%) in 2022 to 6 (35.3%) in 2023 (*p* = 0.018).

### Weather conditions in Hong Kong before, during, and after the melioidosis outbreak

**Figure 4.**
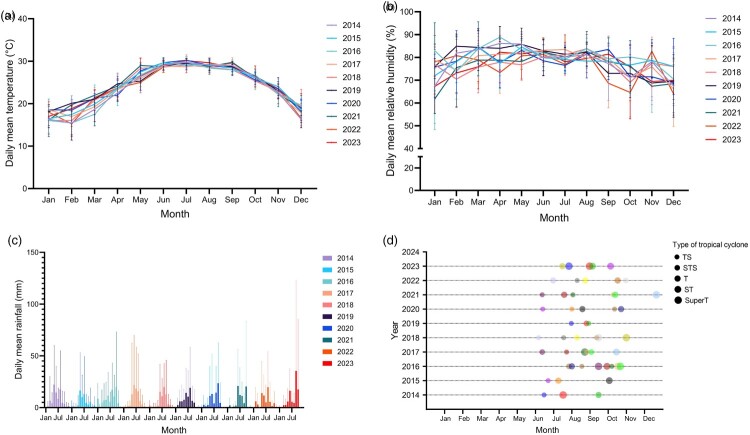
Summary of weather data from Hong Kong, 2014–2023. (A) Daily mean temperature by month. Data represent the monthly mean ± standard deviation (SD). (B) Daily mean relative humidity by month. Data represent the monthly mean ± SD. (C) Daily mean rainfall by month. Data represent the monthly mean + SD. (D) Tropical cyclone warnings of the intensity of tropical storms or above over the years. Each colour represents a different tropical cyclone. Tropical cyclones are classified in accordance with the World Meteorological Organisation's recommendation by their maximum sustained wind speeds near the centre: tropical storm (TS): 63–87 km/h; severe tropical storm (STS): 88–117 km/h; typhoon (T): 118–149 km/h; severe typhoon (ST): 150–184 km/h; super typhoon (SuperT): 185 km/h or above.

The mean daily temperature, relative humidity, total rainfall, and the number of tropical cyclone warning signals are shown in Figure 4. These parameters in the year 2022, during the melioidosis outbreak, showed no significant differences compared to the preceding and subsequent years. Specifically, the daily mean temperature (*p* = 0.30), relative humidity (*p* = 0.91), and rainfall (*p* = 0.97) did not show statistically significant differences between the years 2022 and 2023, when a reduction in the case number of melioidosis was observed. Although there was no statistically significant difference in the weighted tropical cyclone scores between 2022 and 2023 (mean 83.3 vs. 106.1, *p* = 0.44), the increase in score in 2023 supports our hypothesis that the outbreak in 2022 could not be solely attributed to cyclones.

## Discussion

In this study, we investigated the community outbreak of melioidosis in Hong Kong in 2022, which predominantly affected a specific area in the SSP district. We identified *B. pseudomallei* contamination of the soil in the FWSRs that supplied potable water to the SSP district. Phylogenetic analysis demonstrated that both the environmental samples and patient isolates from this district shared the same sequence type (ST-1996) (Figure 2A). Most patient strains were phylogenetically linked to environmental strains collected from FWSR C (Figure 2B). These findings provide supplementary insights into the 2022 melioidosis outbreak, complementing the report by Wu *et al* that highlighted a significant association between melioidosis and the total weighted typhoon signal scores [20]. In their study, *B. pseudomallei* was cultured from one (5%) of the air samples and detected by 16S rRNA gene sequencing in 29.2% of the soil samples collected from nearby building sites and gardening areas, suggesting probable airborne transmission of melioidosis during typhoon seasons [20]. However, with continued monitoring of the weather conditions, there were no apparent changes in adverse weather events between 2022 and 2023, during which a significant reduction in melioidosis cases from SSP district was observed. Therefore, we propose further hypothesis that may explain the 2022 melioidosis outbreak in SPP district, in additional to airborne transmission.

Compared with the study by Wu et al., our investigations revealed a significantly higher detection rate of *B. pseudomallei* from soil samples (10.7% by culture, 53.1% by PCR), especially soil samples collected at the two FWSRs (FWSRs B and C) near the residential buildings of the cases (Supplementary Figure 1). The soil isolates were of the same sequence type as the clinical isolates from patients residing in the SSP region. More importantly, household environmental samples collected at one affected resident’s home were PCR positive for *B. pseudomallei*, indicating that contaminated water supply might be a possible source of this community outbreak. We hypothesize that the episodic seepage of bacteria from the topsoil through minute cracks in the roof of FWSRs could explain this contamination, as evidenced by one roof swab from the inside of FWSRs that tested positive by PCR. Such episodic seepage is more likely to occur during heavy rain when the soil is saturated with water. Although we could not demonstrate the presence of *B. pseudomallei* in the freshwater samples from FWSRs, we cannot exclude the possibility of sampling error due to the massive amount of water in the reservoir and continuous water flow. Furthermore, the contamination might be transient or incidental which could not be detected at the time of sampling. In addition, PCR testing revealed that the swabs of air vents at FWSR B (33.3%) and C (40.0%) were positive for *B. pseudomallei*. This suggests the possibility that soil particles contaminated with *B. pseudomallei* may have entered the FWSRs via the air vent outlets and be indirectly transmitted to patients.

This hypothesis may be questioned by the argument that water supply contamination being the possible cause of this community outbreak was based solely on two weak PCR-positive (culture-negative) samples with low bacterial loads (21.88–44.25 copies/mL) from one patient's home. However, our sampling was limited to a single visit to each residence, while the infected patients had persistent and prolonged exposure to the water supply during activities such as hand washing and bathing. According to a fact sheet published by the U.S. Environmental Protection Agency (EPA) [26], the average shower lasts about 8 min, and with a flow rate of 2.1 gallons per minute. Thus, each shower uses more than 16 gallons of water. In contrast, the volume of freshwater collected for microbiological investigation was 25 mL. In contrast, the daily water exposure during a typical shower is approximately 2,422 times greater than the amount of water collected for culture and PCR testing. Given that the melioidosis outbreak lasted for four months (July–October 2022), the total volume of water exposure for each susceptible household member could reach approximately 7450 L of water. Therefore, negative PCR results from small-volume specimens collected at a single time point after the outbreak was noticed did not necessarily exclude the possibility of exposure to *B. pseudomallei* within household settings.

Another potential criticism of our hypothesis is that the low concentration of bacteria from the contaminated soil may be insufficient to contaminate the chlorinated FWSR and facilitate the spread of the outbreak. We want to emphasize that this low concentration may only affect a very small fraction of the elderly and immunosuppressed population in the affected area (SSP). If the bacterial loads were higher, we would expect to see a higher number of infected cases.

For the sake of prudence, we recommend increasing the residual chlorine level at the water treatment plant outlet to 1.2 ppm. The number of melioidosis cases from SSP district was observed to be significantly decreased from 32 (71.1%) in 2022 to 6 (35.3%) in 2023. While our findings provide an additional hypothesis of the transmission of melioidosis from contaminated soil to household, we cannot completely exclude the possibility of airborne transmission of *B. pseudomallei* as a route of transmission in some, but not all patients. Further research is needed to fully understand the transmission dynamics of melioidosis in the SSP district.

In this outbreak, the exact route of transmission of *B. pseudomallei* is unknown. Given the high positive rate of detection in soil samples and air vents of FWSRs, it cannot be excluded that the bacterium can be transmitted through the airborne route, or may have entered the FWSRs through the mechanisms discussed above. Our findings also raised the possibility of resistance of *B. pseudomallei* to the low-level residual chlorine in FWSRs and downstream domestic water supplies, as shown in previous report [27]. Previous study has demonstrated the variable chlorine tolerance among multiple environmental isolates of *B. pseudomallei* [28].

There are limitations in our study. First, the PCR positive environmental samples collected from the household could not be subjected to genotyping. The *B. pseudomallei* DNA detected could be coming from the patient or dust inside the flat. Second, one possibility for PCR-positive culture-negative environmental samples could be due to non-viable *B. pseudomallei*. A viability assessment was not performed, as it was beyond the scope of this study. Third, quantitative culture was not performed to assess the bacterial load in the environmental samples, nor was soil sampling performed at a predefined depth, which prevented a comprehensive assessment of the variability and extent of soil contamination. Fourth, the volume of freshwater collected for microbiological investigation was small, which may lead to a negative PCR result from the water samples. The implementation of the filtration method for bacterial culture and concentration steps prior to PCR may improve the yield in future investigations. Fifth, readings of residual chlorine levels in water supply downstream of water treatment plants were not available before the outbreak of 2022. In addition, the susceptibility of isolated *B. pseudomallei* strains to chlorine was not determined in vitro, which necessitates further study with serial isolates to demonstrate possible emerging resistance. Sixth, as melioidosis became a notifiable disease only after November 2022, there is a possibility of under-estimation of the number of cases of melioidosis before 2022. Seventh, normal year-to-year variation in melioidosis case numbers may be expected, which may explain the apparent reduction in case number in 2023. Although we did not find a significant difference in the daily mean temperature, relative humidity, and total rainfall between the years 2022 and 2023, July 2022 was the hottest month in Hong Kong since records began in 1884 [29], which was followed by high rainfall in August 2022. Although environmental and climate factors may not necessarily correlate with the incidence of melioidosis in every case, we believe extreme weather conditions might be a contributing factor to the outbreak in July–October 2022 to a certain degree. A prospective study with comprehensive monitoring of environmental contamination is warranted to demonstrate the consistent reduction in case numbers with increased chlorination.

In conclusion, we demonstrated an unusual community outbreak of melioidosis in an urban area of Hong Kong, in which the possibility of *B. pseudomallei* contamination of post-treatment water supply cannot be excluded. Active surveillance and clinical vigilance should be enhanced in areas of high endemicity, and chlorination of water should be maintained and monitored.

## Supplementary Material

Supplementary_File-clean.docx

## References

[1] Limmathurotsakul D, Golding N, Dance DA, et al. Predicted global distribution of *Burkholderia pseudomallei* and burden of melioidosis. Nat Microbiol. 2016 Jan 1;1(1):15008. doi:10.1038/nmicrobiol.2015.8 27571754

[2] Gassiep I, Armstrong M, Norton R. Human melioidosis. Clin Microbiol Rev. 2020 Mar 11;33(2):e00006–19. doi:10.1128/CMR.00006-19 32161067 PMC7067580

[3] Meumann EM, Limmathurotsakul D, Dunachie SJ, et al. *Burkholderia pseudomallei* and melioidosis. Nat Rev Microbiol. 2024 Mar;22(3):155–169. doi:10.1038/s41579-023-00972-5 37794173

[4] Liu X, Pang L, Sim SH, et al. Association of melioidosis incidence with rainfall and humidity, Singapore, 2003-2012. Emerg Infect Dis. 2015 Jan;21(1):159–162. doi:10.3201/eid2101.140042 25531547 PMC4285244

[5] Currie BJ, Jacups SP. Intensity of rainfall and severity of melioidosis, Australia. Emerg Infect Dis. 2003 Dec;9(12):1538–1542. doi:10.3201/eid0912.020750 14720392 PMC3034332

[6] Mu JJ, Cheng PY, Chen YS, et al. The occurrence of melioidosis is related to different climatic conditions in distinct topographical areas of Taiwan. Epidemiol Infect. 2014 Feb;142(2):415–423. doi:10.1017/S0950268813001271 23714119 PMC9151114

[7] Lui G, Tam A, Tso EYK, et al. Melioidosis in Hong Kong. Trop Med Infect Dis. 2018;3(3):91. doi:10.3390/tropicalmed3030091 30274487 PMC6161032

[8] The Government of the Hong Kong Special Administrative Region. CHP appeals for heightened vigilance against melioidosis infection [updated on October 12, 2022; accessed 2024 April 26]. Available from: https://www.info.gov.hk/gia/general/202210/12/P2022101200569.htm.

[9] National Notifiable Diseases Surveillance System (NNDSS). Melioidosis (*Burkholderia pseudomallei*) 2023 case definition. [accessed 2024 March 10]. Available from: https://ndc.services.cdc.gov/case-definitions/melioidosis-burkholderia-pseudomallei/.

[10] Wong SCY, Wong SC, Chen JHK, et al. Polyclonal *Burkholderia cepacia* complex outbreak in peritoneal dialysis patients caused by contaminated aqueous chlorhexidine. Emerg Infect Dis. 2020 Sep;26(9):1987–1997. doi:10.3201/eid2609.191746 32818396 PMC7454066

[11] Cheng VCC, Wong SC, Chen JHK, et al. Control of multidrug-resistant *Acinetobacter baumannii* in Hong Kong: role of environmental surveillance in communal areas after a hospital outbreak. Am J Infect Control. 2018 Jan;46(1):60–66. doi:10.1016/j.ajic.2017.07.010 28893447

[12] Wong SC, Lam GK, Chen JH, et al. Air dispersal of multidrug-resistant *Acinetobacter baumannii*: implications for nosocomial transmission during the COVID-19 pandemic. J Hosp Infect. 2021 Oct;116:78–86. doi:10.1016/j.jhin.2021.08.005 34403765 PMC8429036

[13] Cheng VC, Wong SC, Chan VW, et al. Air and environmental sampling for SARS-CoV-2 around hospitalized patients with coronavirus disease 2019 (COVID-19). Infect Control Hosp Epidemiol. 2020 Nov;41(11):1258–1265. doi:10.1017/ice.2020.282 32507114 PMC7327164

[14] Cheng VCC, Wong SC, Chen JHK, et al. Escalating infection control response to the rapidly evolving epidemiology of the coronavirus disease 2019 (COVID-19) due to SARS-CoV-2 in Hong Kong. Infect Control Hosp Epidemiol. 2020 May;41(5):493–498. doi:10.1017/ice.2020.58 32131908 PMC7137535

[15] Lau SK, Tang BS, Curreem SO, et al. Matrix-assisted laser desorption ionization-time of flight mass spectrometry for rapid identification of *Burkholderia pseudomallei*: importance of expanding databases with pathogens endemic to different localities. J Clin Microbiol. 2012 Sep;50(9):3142–3143. doi:10.1128/JCM.01349-12 22718946 PMC3421815

[16] Cunningham SA, Patel R. Importance of using Bruker's security-relevant library for Biotyper identification of Burkholderia pseudomallei, Brucella species, and Francisella tularensis. J Clin Microbiol. 2013;51(5):1639–1640. doi:10.1128/JCM.00267-13 23447635 PMC3647932

[17] Woo PC, Leung PK, Tsoi HW, et al. Characterization of a novel insertion sequence, IS Bp1, in *Burkholderia pseudomallei*. Arch Microbiol. 2002 Mar;177(3):267–273. doi:10.1007/s00203-001-0389-8 11907683

[18] Warnings & Signals Database, The Hong Kong Observatory. 2024. [accessed 2024 March 9]. Available from: https://www.hko.gov.hk/en/cis/warndb.htm.

[19] Hong Kong Observatory. Monthly Meteorological Normals for Hong Kong (1991–2020). [accessed 2024 April 11]. Available from: https://www.hko.gov.hk/en/cis/normal/1991_2020/normals.htm.

[20] Wu WG, Shum MH, Wong IT, et al. Probable airborne transmission of *Burkholderia pseudomallei* causing an urban outbreak of melioidosis during typhoon season in Hong Kong, China. Emerg Microbes Infect. 2023 Dec;12(1):2204155. doi:10.1080/22221751.2023.2204155 37070526 PMC10155638

[21] Su HP, Chan TC, Chang CC. Typhoon-related leptospirosis and melioidosis, Taiwan, 2009. Emerg Infect Dis. 2011 Jul;17(7):1322–1324. doi:10.3201/eid1707.101050 21762606 PMC3381404

[22] Census and Statistics Department. The Government of the Hong Kong Special Administrative Region. District Profiles (Population and Households) 2023. [accessed 2024 July 19]. Available from: https://www.censtatd.gov.hk/en/map_ghs.html.

[23] Gee JE, Bower WA, Kunkel A, et al. Multistate outbreak of melioidosis associated with imported aromatherapy spray. N Engl J Med. 2022 Mar 3;386(9):861–868. doi:10.1056/NEJMoa2116130 35235727 PMC10243137

[24] The Government of the Hong Kong Special Administrative Region. LCQ10: Use of the space on the rooftops of service reservoirs [updated July 4, 2018; accessed 2024 April 26]. Available from: https://www.info.gov.hk/gia/general/201807/04/P2018070400776.htm.

[25] The Government of the Hong Kong Special Administrative Region. Government gazettes inclusion of melioidosis as statutorily notifiable infectious disease under Prevention and Control of Disease Ordinance [updated November 11, 2022; accessed 2024 April 26]. Available from: https://www.info.gov.hk/gia/general/202211/11/P2022111100374.htm.

[26] United States Environmental Protection Agency. Save water and energy by showering better [accessed 2025 January 18]. Available from: https://www.epa.gov/sites/default/files/2017-02/documents/ws-ourwater-shower-better-learning-resource_0.pdf.

[27] Howard K, Inglis TJ. The effect of free chlorine on *Burkholderia pseudomallei* in potable water. Water Res. 2003 Nov;37(18):4425–4432. doi:10.1016/S0043-1354(03)00440-8 14511713

[28] O’Connell HA, Rose LJ, Shams A, et al. Variability of *Burkholderia pseudomallei* strain sensitivities to chlorine disinfection. Appl Environ Microbiol. 2009 Aug;75(16):5405–5409. doi:10.1128/AEM.00062-09 19542324 PMC2725453

[29] Hong Kong Observatory. Monthly Weather Summary [accessed on April 11, 2024]. Available from: https://www.hko.gov.hk/en/wxinfo/pastwx/mws/mws.htm.

