# Supplementary material for: Outbreak of melioidosis in a highly urbanized area
Source: Emerg Microbes Infect. 2025 Jul 24;14(1):2539194. doi: 10.1080/22221751.2025.2539194 (PMC12340941; doi:10.1080/22221751.2025.2539194)
Supplement: Supplementary_File-clean.docx [file TEMI_A_2539194_SM5727.docx]

**SUPPLEMENTARY FILE**

| **Supplementary Table 1** | Yearly case number and geographical distribution of melioidosis cases in Hong Kong, 2014–2023 |
| --- | --- |
| **Supplementary Table 2** | Annual incidence of melioidosis cases in Hong Kong, 2014-2023 |
| **Supplementary Table 3** | Cumulative incidence of melioidosis cases across districts in Hong Kong, 2014–2023 |
| **Supplementary Table 4** | Ct value distribution of PCR-positive environmental samples |
| **Supplementary Table 5** | Average read count for each isolate included in Figure 2 |
| **Supplementary Figure 1** | Location of the freshwater service reservoirs (FWSRs) included in environmental investigation |
| **Supplementary Figure 2** | The design of air vents on top of the freshwater service reservoirs |

Supplementary Table 1. Yearly case number and geographical distribution of melioidosis cases in Hong Kong, 2014–2023

| Year | CW | Ea | Is | KC | KTs | KTo | SK | ST | SSP | So | TP | TM | WTS | YTM | YL | N/A | Total |
| --- | --- | --- | --- | --- | --- | --- | --- | --- | --- | --- | --- | --- | --- | --- | --- | --- | --- |
| 2014 |  |  | 1 |  |  |  | 1 |  |  | 3 |  |  | 1 |  |  |  | 6 |
| 2015 |  |  |  |  |  |  |  | 1 | 1 |  |  | 1 |  |  |  |  | 3 |
| 2016 |  |  | 1 | 2 |  | 1 |  |  | 1 |  |  |  |  |  |  |  | 5 |
| 2017 |  | 3 | 2 | 2 |  |  | 1 | 1 | 1 | 1 |  |  |  |  |  |  | 11 |
| 2018 | 1 |  | 1 | 2 | 1 |  |  |  |  |  |  |  |  |  |  | 1 | 6 |
| 2019 |  |  |  |  |  |  |  |  | 2 |  | 1 |  |  |  |  |  | 3 |
| 2020 |  | 1 |  |  | 1 | 1 |  |  | 3 | 1 | 1 | 1 |  | 1 | 1 |  | 11 |
| 2021 |  | 3 |  | 3 |  | 3 | 2 |  | 3 |  |  |  |  | 1 |  |  | 15 |
| 2022 |  | 3 |  | 1 | 1 | 2 | 2 | 1 | 32 |  |  |  | 2 | 1 |  |  | 45 |
| 2023 |  | 3 | 2 |  | 1 | 1 |  |  | 6 | 1 | 1 |  | 1 |  |  | 1 | 17 |
| Total | 1 | 13 | 7 | 10 | 4 | 8 | 6 | 3 | 49 | 6 | 3 | 2 | 4 | 3 | 1 | 2 | 122 |

Abbreviations: CW: Central & Western; Ea: Eastern; Is: Islands; KC: Kowloon City; KTs: Kwai Tsing; KTo: Kwun Tong; SK: Sai Kung; ST: Sha Tin; SSP: Sham Shui Po; So: Southern; TP: Tai Po; TM: Tuen Mun; WTS: Wong Tai Sin; YTM: Yau Tsim Mong; YL: Yuen Long; N/A: not available.

Supplementary Table 2. Annual incidence of melioidosis cases across districts in Hong Kong, 2014-2023

| **Year** | **Total No. of cases** | **Hong Kong population** | **Incidence (per 100,000 population)** |
| --- | --- | --- | --- |
| 2014 | 6 | 7,252,900 | 0.08 |
| 2015 | 3 | 7,309,700 | 0.04 |
| 2016 | 5 | 7,378,100 | 0.07 |
| 2017 | 11 | 7,414,800 | 0.15 |
| 2018 | 6 | 7,487,700 | 0.08 |
| 2019 | 3 | 7,520,500 | 0.04 |
| 2020 | 11 | 7,426,700 | 0.15 |
| 2021 | 15 | 7,401,500 | 0.20 |
| 2022 | 45 | 7,472,600 | 0.60 |
| 2023 | 17 | 7,527,900 | 0.23 |

Supplementary Table 3. Cumulative incidence of melioidosis cases across districts in Hong Kong, 2014–2023

| **District** | **Total No. of cases, 2014-2023** | **Land-based non-institutional population, 2023 [1]** | **Incidence (per 100,000 population)** |
| --- | --- | --- | --- |
| Sham Shui Po | 49 | 434,100 | 11.3 |
| Eastern | 13 | 520,200 | 2.5 |
| Kowloon City | 10 | 411,800 | 2.4 |
| Kwun Tong | 8 | 666,500 | 1.2 |
| Islands | 7 | 195,700 | 3.6 |
| Sai Kung | 6 | 503,000 | 1.2 |
| Southern | 6 | 255,900 | 2.3 |
| Kwai Tsing | 4 | 490,400 | 0.8 |
| Wong Tai Sin | 4 | 403,800 | 1.0 |
| Sha Tin | 3 | 700,300 | 0.4 |
| Tai Po | 3 | 314,800 | 1.0 |
| Yau Tsim Mong | 3 | 304,200 | 1.0 |
| Tuen Mun | 2 | 532,000 | 0.4 |
| Central & Western | 1 | 232,500 | 0.4 |
| Yuen Long | 1 | 670,000 | 0.1 |
| N/A | 2 | - | - |
| Total | 122 | 7,447,700 | 1.6 |

Supplementary Table 4. Ct value distribution of PCR-positive environmental samples

| **Specimen type** | **Location** | **Percentage positive** | **Mean Ct value (range)^*^** |
| --- | --- | --- | --- |
| Soil, lawn/ground | FWSR A | 19.0% (4/21) | 14.32 (11.60–16.39) |
|  | FWSR B | 63.6% (7/11) | 14.46 (7.63–19.74) |
|  | FWSR C | 76.7% (23/30) | 11.56 (6.36–20.14) |
|  | FWSR D | 9.1% (2/22) | 16.23 (14.06–18.39) |
|  | FWSR E | 45.0% (9/20) | 13.83 (11.55–15.44) |
|  | Total | 43.3% (45/104) | 12.92 (6.36–20.14) |
| Soil, corners of air vents | FWSR A | 57.5% (23/40) | 13.47 (7.83–16.98) |
|  | FWSR B | 100.0% (20/20) | 12.41 (4.75–19.10) |
|  | FWSR C | 85.0% (17/20) | 13.17 (5.92–16.73) |
|  | FWSR D | 35.0% (14/40) | 12.63 (9.90–15.86) |
|  | Total | 61.7% (74/120) | 12.98 (4.75–19.10) |
| Air vent swabs | FWSR B | 33.3% (3/9) | 15.70 (13.47–17.89) |
|  | FWSR C | 40.0% (2/5) | 17.10 (16.69–17.50) |
|  | Total | 11.1% (5/45) | 16.26 (13.47–17.89) |
| Roof swabs | FWSR B | 4.8% (1/21) | 15.28 |

Abbreviations: FWSR: freshwater service reservoir.

^*^By nested real-time PCR assay.

Supplementary Table 5. Average read count for each isolate included in Figure 2*

| **Sample name** | **No of reads (R1+R2)** |
| --- | --- |
| QMH13 | 8713486 |
| QMH18 | 9223766 |
| QMH19 | 8768068 |
| QMH33 | 8632784 |
| RF22000096 | 3496760 |
| RF22000138 | 3084242 |
| RF22000160 | 3369116 |
| RF22000290 | 3465120 |
| RF22000403 | 3190160 |
| RF22000422 | 4865420 |
| RF22000508 | 4135568 |
| RF22000557 | 4526748 |
| RF22000622 | 6483338 |
| RF22000627 | 8820908 |
| RF22000635 | 5295886 |
| RF22000636 | 6273786 |
| RF22000647 | 5449508 |
| RF22000648 | 3514298 |
| RF22000654 | 3471466 |
| RF22000659 | 4659976 |
| RF22000662 | 6625420 |
| RF22000725 | 4032302 |
| RF22000732 | 2764278 |
| RF22000757 | 6315044 |
| RF22000761 | 2627208 |
| RF22000773 | 3322094 |
| RF22000778 | 3460066 |
| RF22000787 | 3133636 |
| RF22000801 | 3536222 |
| RF22000802 | 9917596 |
| RF22000850 | 3180300 |
| RF22000855 | 3350918 |
| RF22000856 | 3301516 |
| RF22000861 | 3100256 |
| RF22000867 | 5079402 |
| RF22000868 | 5731620 |
| RF22000917 | 3576700 |
| RF22000924 | 3559248 |
| RF22000935 | 2905666 |
| RF22000936 | 2487728 |
| RF22000937 | 3000416 |
| RF22000938 | 3303962 |
| RF22000939 | 3110834 |
| RF22000940 | 3137336 |
| RF22000941 | 3595324 |
| RF22000942 | 3214600 |
| RF22000943 | 3300770 |
| RF22000944 | 3883610 |
| RF22000945 | 3325158 |
| RF22000953 | 4686584 |
| RF22000956 | 3874708 |
| RF22000957 | 3012632 |
| RF22000958 | 3224854 |
| RF22000959 | 2210416 |
| RF22000960 | 3230572 |
| RF22000961 | 2795876 |
| RF22000968 | 3778502 |
| RF22001005 | 7238452 |
| RF22001008 | 7749922 |
| RF22001009 | 8246592 |
| RF22001020 | 5092698 |
| RF22001026 | 7127036 |
| RF22001027 | 7306316 |
| RF22001028 | 7060092 |
| RF22001029 | 4830716 |
| RF22001039 | 5442948 |
| RF22001071 | 6959364 |
| RF22001072 | 6205686 |
| RF22001073 | 7057646 |
| RF23000026 | 5251732 |
| RF23000173 | 4792540 |
| RF23000251 | 4752310 |
| RF23000291 | 3755956 |
| RF23000321 | 4983646 |
| RF23000601 | 3229002 |
| RF23000771 | 2643962 |
| RF23000822 | 2926290 |
| RF23000874 | 3539172 |
| RF23000895 | 3037414 |
| RF23000940 | 3151992 |
| RF23001028 | 2801870 |
| RF23001109 | 2334580 |
| RF23001146 | 3638520 |

^*^ Average read count (R1+R2) : 4,587,138 reads.

Supplementary Figure 1. Location of the freshwater service reservoirs (FWSRs) included in environmental investigation. The FWSRs are shaded in indigo. The five FWSRs (A to E) are marked with red letters. The district of Sham Shui Po (SSP) is shaded in lilac. Image was created using ArcGIS Online (Esri).


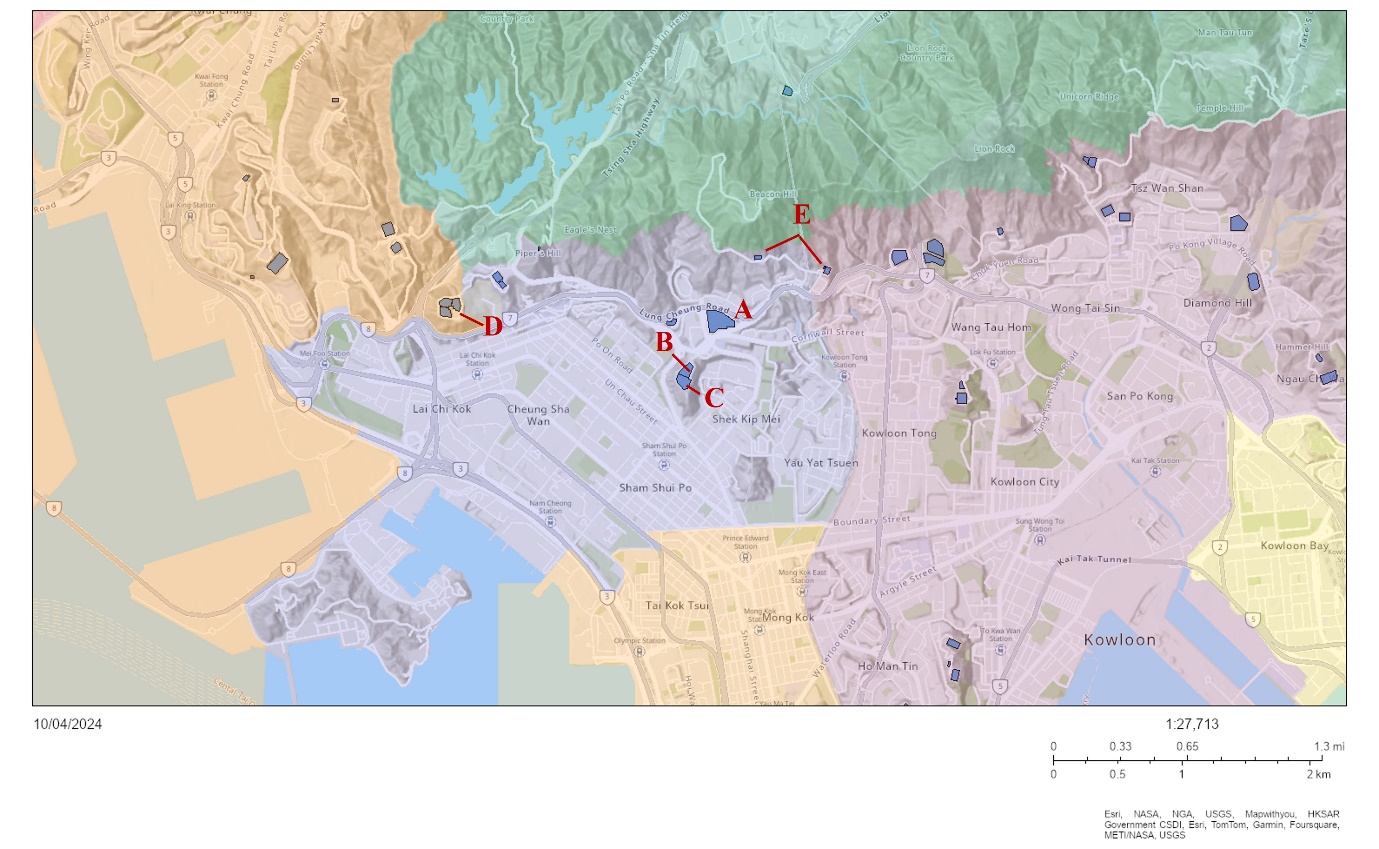


Supplementary Figure 2. The design of air vents on top of the freshwater service reservoirs. A. Schematic diagram of air vent design. Air vents with wire gauze mesh filters were installed at the top of the reservoirs and elevated above the ground. It equalised the pressure difference between the outside and the inside of the enclosed concrete reservoir, where negative pressure was generated by water drainage. The wire gauze mesh filters were designed to prevent the ingress of large-particle foreign substances. B. Photo of an air vent with cover removed.


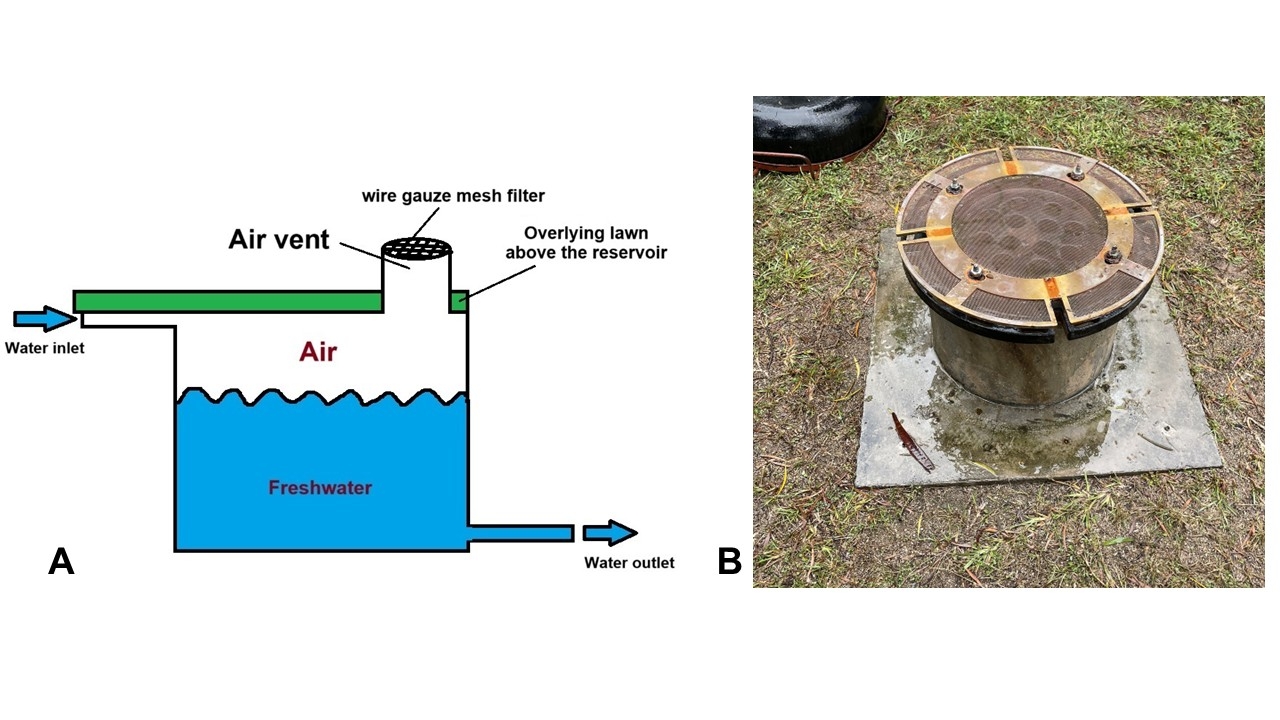


**References**

1. Census and Statistics Department, The Government of the Hong Kong Special Administrative Region. District Profiles (Population and Households) 2023. [Accessed on July 19, 2024] Available from: https://www.censtatd.gov.hk/en/map_ghs.html.
